# Practices and attitudes of herbalists regarding informed consent in Uganda: A qualitative study

**DOI:** 10.21203/rs.3.rs-3911823/v1

**Published:** 2024-02-08

**Authors:** Sumayiya Nalubega, Paul Kutyabami, Adeline Twimukye, David. K. Mafigiri, Nelson. K. Sewankambo

**Affiliations:** The AIDS Support Organisation; Makerere University; Makerere University; Makerere University Kampala; Makerere University

**Keywords:** Herbal medicine, informed consent, communal consent, ethical principles

## Abstract

**Background:**

Informed consent (IC) is a fundamental principle in medical ethics that upholds respect for patient autonomy. Although widely applied in healthcare, its feasibility and implementation in herbal medicine have been underexplored. This study therefore aimed to explore the practices and attitudes of herbalists regarding informed consent.

**Methods:**

To achieve these objectives, a qualitative cross-sectional study was conducted from June to December 2020. Twenty-one in-depth interviews with herbalists and four key informant interviews with leaders of the different traditional medicine organizations were also conducted. The data were analyzed thematically using NVivo version 12 software.

**Results:**

Sixteen of the twenty-one participants acquired oral herbal medicine knowledge from their relatives. Although a positive inclination toward obtaining IC was evident, the focus was on disclosing basic information. Discussions of alternative treatments and herbal specifics less frequent. Disease management decisions often involve shared responsibility within families or societies. Documented IC procedures are rare among herbalists, who deem consent forms unnecessary, although they recognize the potential benefits of IC in fostering trust and professionalism. Challenges hindering IC implementation included regulatory gaps, inadequate skills, and the absence of mechanisms to protect the intellectual property rights of herbal medicine.

**Conclusion:**

This study illuminates how educational, cultural, familial, and regulatory factors influence herbalists’ practices and attitudes toward informed consent.

## BACKGROUND

In medical ethics, informed consent (IC) is an ethical imperative that reflects respect for a person’s autonomy and right to self-determination. (1–3) and the fiduciary responsibilities of the patient and healthcare provider (4, 5). The IC process involves providing patients with sufficient information for autonomous decision-making, ensuring clarity and freedom from coercion (6, 7). Essential information includes details about the procedure, benefits, risks, alternative treatments, and treatment costs (8). While IC is well established in conventional medicine, questions arise about its compatibility with traditional medicine (TM), which is prevalent in developing countries such as Uganda, where 70–80% of the population relies on traditional healers, particularly herbalists (9, 10). Herbal medicines in Uganda are generally sold by herbalists with little or no formal education or in-service training in IC or medical ethics (11). The ability of practitioners to effectively execute IC has been shown to be associated with having had some training in this topic or medical ethics (7). A study by Caspi et al reported variance and a lack of standards in the conduct of IC practices among complementary and alternative medicine (CAM) therapies (12), and these practices have been reported to vary based on different jurisdictions and cultures. This study aimed to investigate the knowledge gap among herbalists by exploring and documenting their practices and attitudes regarding IC implementation in Uganda.

## METHODS

### Study Design

This study had a cross-sectional design and used in-depth interviews (IDIs) and key informant interviews (KIIs) to collect the data. The design and method of data collection were chosen because the team needed to attain a deeper understanding of the practices and attitudes of the herbalists regarding the use of informed consent.

### Study setting

The study was conducted in the Wakiso and Mpigi districts through PROMETRA Uganda, a nongovernmental organization (NGO) that promotes TM and the preservation of indigenous traditional knowledge, including herbal medicine. These districts were chosen because they host institutions that train, educate and bring together herbalists from different parts of the country and have a number of traditional medicine practitioners.

### Sampling Procedure

Participants for the 21 IDIs were identified and purposively selected based on their specialty and expertise in herbal medicine from PROMETRA Uganda. Herbalists who were unreachable by phone were visited at their premises to schedule appointments for the interviews. Four key informants were recruited from different traditional medicine organizations responsible for coordinating traditional medical practitioners within the Wakiso and Mpigi districts. These included the National Chemotherapeutics Institute (NACRI), Uganda Nédaggala Lyayo, PROMETRA, and the National Council for Traditional Healers and Herbalists Associations (NACOTHA). These were contacted through phone calls for appointments regarding the dates and times they would be available for the interviews. Interviews were conducted from silent places chosen by the participants to ensure that the recording and confidentiality of the participants’ information were not interrupted.

### Data collection

Data collection was conducted from June to December 2020 by a team of two people: the principal investigator, who holds a Master of Health Sciences degree in Bioethics, and a Bachelor of Science majoring in Biochemistry. The second, who was a research assistant, holds a Master of Health Sciences degree in bioethics and a Bachelor of Social Sciences with a focus on political science. Both of them had over two years of experience and training in qualitative data collection methods, which included conducting in-depth interviews and key informant interviews, as well as obtaining informed consent from potential participants.

### Data collection tool

An interview guide comprising questions assessing herbalists’ practices and attitudes toward informed consent, based on the elements of IC, such as assessing patients’ competency, information disclosure, voluntariness, understanding, and consent (decision and authorization), was utilized to conduct the interviews. Participants were individually informed about the study in silent rooms, given time to ask questions, and freely agreed to participate and signed informed consent forms. These interviews were conducted in Luganda, a local language preferred by all participants who could read and fully comprehend it. The interviews, which lasted between 40 and 90 minutes, were audio-recorded, transcribed verbatim, translated into English, and analyzed thematically. In total, 21 IDIs and four key KIIs were detected.

#### Data analysis

All the data were analyzed thematically using NVivo (version 12) software. A coding framework based on six transcripts that were manually reviewed and coded to generate the initial set of codes was developed. All transcripts were imported into NVivo software, and open coding was performed. Two members of the research team (SN and AT) independently reviewed the interviews and created inductive codes, which were organized in a codebook. SN and AT discussed and resolved inconsistencies in coding, and the final codes were established by consensus between the coders. The coded text was then categorized into themes. Illustrative quotations for each emergent theme were selected for results narration. The study followed the COREQ checklist (13) for reporting and analyzing the data.

## RESULTS

### Demographics

In this study, demographic data were collected from 25 study participants (21 herbalists who underwent in-depth interviews and four herbalists’ association leaders who were key informants). There were 15 females (median age of 54 years), and 14 had attained primary education only. Ten herbalists had more than 20 years of experience in their field. A summary of the demographics of the 21 in-depth interviewees is presented in Table 1. The association leaders of the four herbalists were all males aged 30 to 60 years. All four leaders held a bachelor’s degree and had more than five years of working experience as herbalists.

Analysis of the data yielded five themes: description of an herbalist and source of herbal medicine knowledge, attitudes of herbalists toward the IC, practices of obtaining IC, factors that prompted them to obtain IC, and barriers that hindered them from practicing IC adequately.

### Description of the herbalist

The participants described herbalists in the following categories: (i) individuals who treat patients’ diseases using naturally occurring plants, including their leaves, flowers, seeds, stems and roots; (ii) individuals who do not attend to patients but teach other people herbal medicines; and (iii) individuals who collect and process herbs from forests and gardens but do not directly attend to patients. These patients were further categorized into different specialties, such as traditional birth attendants, bone setters, and dispensers;

“I am looking at an herbalist as an individual who is approached by a patient, examines their health status and, depending on what the patient explains to them, picks certain herbs, roots, leaves, stems or any plant material, mix and prepare them to treat the patients’ ailment.” (KII_Male_4)

A ‘herbalist is one who treats diseases using naturally occurring elements such as trees and herbs that were created by God” (IDI_Female_04).

### Source of knowledge regarding herbal medicine

The majority of participants reported acquiring herbal knowledge orally and informally from their relatives. They noted that herbalism has a robust foundation in traditional healing. Some participants had also undergone formal training in herbalism schools, learned from colleagues, and gained knowledge and experience through long-term use of various herbs for a range of diseases, such as malaria, skin diseases, diarrhea, and sexually transmitted diseases. Patients with chronic diseases also provided antenatal care services and managed the side effects of long-term drug medication.

“I got this knowledge from my grand-parents, then from my father who used to work with Nakalooli brothers in Kisubi.” (IDI_Male_12)

“There is a category of herbalists who have undergone training and been educated. They go to medical schools or natural chemotherapeutics, and they learn about the herbs; there are some who read from the internet and start practicing”. (KII_Male_4)

### Herbalists’ attitudes toward informed consent

The majority of the study participants regarded ICs as essential for adequately disclosing information about the proposed treatment to the patient and for identifying the misconceptions people have about herbal medicine (safety and side effects). The authors suggested that disclosure should include treatment benefits, costs, dosage, and side effects.

“it is beneficial to inform the patient about the medicine’s benefits, risks, and potential side effects to ensure they leave satisfied, confident, and well informed based on the explanation provided. Nevertheless, there are instances when withholding certain information becomes necessary particularly if revealing everything might induce fear and panic in the patient, potentially exacerbating their problems.” (IDI_Female_09)

“While there is a common belief that these herbs are entirely safe and natural, there are instances where improper consumption can lead to harm. Thus, it is essential to clarify to patients what they should anticipate after taking the medication.”(IDI_Female_05)

“it is very good practice to explain to the patient thoroughly what the treatment entails, such that they can make valid and informed decisions.” (IDI_Male_13)

However, there was nearly unanimous agreement among them that revealing information about alternative treatments and the specific components of herbal mixtures was not necessary for patients, and they believed it could have negative financial and professional implications for them.

“Some individuals, upon learning about the herbs used in a formulation, may falsely claim to be herbalists without understanding the proper preparation, usage, dosage, and comprehensive applications. Subsequently they may misinform others, attributing their newfound knowledge to the original herbalist. This is why I limit the information I share with patients to avoid potential harm to my profession and reputation from unqualified individuals misrepresenting themselves as herbalists. ” (IDI_Female_005)

“I do not reveal the specific herbs I blend to create the formulation. Instead, I focus on preparing the most effective medicine tailored to treating the patient’s specific ailment. This approach eliminates the need for the patient to seek alternative treatments, as I consistently provide the best treatment within my knowledge” (IDI_Female_007)

The study participants did not perceive it necessary to inquire whether patients voluntarily accepted or refused the suggested treatment. The patients’ arrival at the herbalists’ premises was automatically construed as their acceptance of the impending treatment. Consequently, the herbalists did not inquire about their patients’ perspectives regarding the treatment offered by the herbalist.

“Only the herbalist is allowed to make decisions and not the patient; patients do not tell us what to do, and therefore, they take whatever we as herbalists choose (Kll_Male-1).

“A patient is not supposed to make any decisions because he or she does not have knowledge regarding the treatment. (IDI_Female_02)

Some herbalists emphasized the significance of shared decision-making between themselves and the patient because this approach allowed for mutual advice and education during discussions, and the herbalist did not possess an absolute right to make decisions for the patient.

“The herbalist should not assume to know everything and decide alone. Patients may possess knowledge that the herbalist lacks. We need to collaborate and engage in discussions, and I as the herbalist, must inquire whether the patient accepts or rejects the suggested treatment. It is the herbalist’s responsibility to educate the patient ensuring they are informed about their choices” (IDI_Female_04)

“You have to explain to the patient; you then give them a chance to decide for themselves either to accept or refuse the treatment.” (IDI_Female_09)

The participants did not believe that signing consent forms was necessary. Moreover, some herbalists were unable to write, making it impractical for them to sign such forms. The participants viewed signing consent forms as irrelevant to herbal medicine and time consuming and asserted that the significance of the consent forms was associated with the use of conventional medicine for administrative purposes.

“The patients trust us; therefore, there is no need for the patient to sign anywhere because by the time they approach you for medicine, they trust you…… Usually, there is no time to ask much or even sign consent forms.” (IDI_Female_09)

#### Informed consent was obtained from the herbalists

The herbalists who disclosed information reported that they needed to disclose the information to patients since they regarded themselves as having more information and knowledge regarding treatment than did the patients. At times, the information shared was based on the kind of questions asked by the patients. The information shared by almost all study participants included common herbal side effects, the cost of medicine and the dosage of the formulation provided, dietary restrictions and any known drug interactions with the herbs provided.

“You clarify the risks linked to the formulation, emphasizing that some individuals erroneously believe herbal medicine is devoid of side effects or the possibility of overdosing, despite the reality that it does have both side effects and the potential for overdoses” (IDI_Female_02)

“I tell the patient the cost of the treatment before I prepare the medicine, such that we can agree. I also tell them about the dosage, and I encourage them to first eat food before taking the medicine.” (IDI_Female_12)

“I further teach them about diet, encourage them to eat vegetables and roughage, not to drink sugar in case they are diabetic. I generally give them health education, and it is that which consumes most of our time” (IDI_Male_16)

Some participants refrained from disclosing the patient’s diagnosis if they anticipated family conflict or increased stress. Additionally, for some illnesses perceived not as an individual’s burden but rather as a communal or family matter, the latter often take precedence in receiving information about the patient’s diagnosis and making treatment decisions about the patients themselves.

Another consideration that hindered study participants from disclosing their diagnosis to them was the fear of self-medication following media advertisements about different herbs.

“Now, if we talk about informed consent from a communal perspective, in our culture and setting, illness is not an individual person’s issue but rather a community or family concern. The patient’s family asks you not to disclose information to the patient; rather, all the information and decisions are made by them.” (KII_Male_4)

In addition, herbalists considered a patient’s capacity to comprehend information to determine whether to initiate discussions, which in turn determined their decision-mating practices. For instance, guardians or parents may engage in discussions and decision making for the child’s treatment. Similarly, for very ill patients, a designated proxy, often a family member, was furnished with the information and assumed the responsibility of making decisions regarding the patient’s treatment choices.

### Assessment of patients’ understanding of the information given

Participants highlighted various methods for assessing patient understanding of provided information. This evaluation involved utilizing questions, interpreting body language, and conducting follow-up phone calls to gauge adherence to prescribed treatments. However, some participants neglected to employ any verification methods to confirm patient comprehension, placing less importance on whether patients understood the shared information, as long as discussions centered around treatment dosages.

“I rephrase a question… I paraphrase it again and ask it again to be sure that the patient has understood what I have told them.” (KII__Maie_2)

‘I do not know how. I just explain and leave the rest to God, for him to have mercy on the patient and help him/her understand and remember whatever you have told them. “(IDI_Female_03)

### Documentation of informed consent

Participants emphasized a practice founded on trust, honesty, and voluntarism. The majority of the studies solely recorded patients’ personal details, medical history, and prescribed medication. Informal communication typically determines treatment acceptance or refusal. Patients did not sign any informed consent documents but provided implied consent through verbal agreement to take the medicine. Additionally, some herbalists lacking writing skills could not provide documented informed consent. A key informant mentioned that, despite being considered necessary in medical ethics, informed consent documentation is not applied in herbal medicine since neither the patient nor the herbalist deems it necessary.

“I have never let a patient sign anywhere. However we agree verbally; they then take the medication…… and imply they have accepted.” (IDI _female_02)

“Some of us don’t know how to write, and we also do not see the importance of making the patient sign those consent forms. I don’t know whether any herbalists practice written informed consent.” (KIL_Male_4)

“We have a registry book and referral forms; however, we do not have consent forms on which patients signed. It is usually a verbal consent.” (IDI_Female_05)

Notably, a few of the participants reported documenting consent for patients or relatives, especially for patients with a terminal illness such as cancer, to avoid blame in case the patient died. Finally, there is a need for evidence in case queries from relatives on the treatment presented at some point.

‘Some patients arrive critically ill with cancer. After administering medication, they are at risk of succumbing, and if that occurs, accusations may arise linking their deaths to the prescribed medicine. To address such situations, consent forms are signed, and I keep a copy along with the patient’s relative during this process.’ (KII_Male_1)

“I document whether the patient accepted or declined the treatment, though the patient themselves does not sign or write anything. This practice ensures that when their relatives visit, I can promptly provide assistance. It also serves as confirmation that I provided treatment at my center.” (IDI_Female-20)

### Factors that facilitate the practice of IC among herbalists

For participants who practiced and perceived informed consent to be important, this was because they perceived it to be a way to enhance the good relationship between the patient and the herbalist, to attract more patients, to portray trust and honesty, which enhanced the good relationship between the patient and the herbalist.

“it is very good as it enables you to establish a good relationship with the patient such that they can easily open up to you, create awareness of whatever is going on, avoid stressing the patient unnecessarily, and know that they have come to the right person and place.” (IDI_FemaleI_18)

Some participants felt that IC would help them avoid blame and false accusations from patients or blame by the public and relatives if a sick person experienced harm after receiving medicine. In this case, documenting consent was considered a protective measure against potential liability.

The correct procedure would have been to document and sign informed consent forms (ICFs), but we have not been following it. The patient might accuse you of providing medication that caused harm, even if they had visited another herbalist who gave them the medicine that led to harm. With proper documentation, you are protected from false accusations and blame. (IDI_Male_11)

“Disclosure and discussion of side effects with the patients is the only thing that can protect the herbalist from being sued in court because they can clearly confirm that they informed the patients of the side effects before they dispensed it to them. (KII_Male_02)

Most participants reported that sharing information and proper explanations during the consent process would help patients adhere to the treatment, restore hope and hence improve treatment outcomes.

“It helps a lot because the patient gets to know that you are honest and trustworthy, you understand what you are doing… such that they are confident about the treatment you give them that they will be well.” (IDI_Female_17)

Practicing IC was perceived as a sign of transparency and professionalism among providers and ethically added value to their job and ensured the safety of their patients.

“If you don’t tell them, you will have done injustice to them. You need to tell the patient because some women come in while pregnant; this means that there are certain kinds of herbs you cannot give them, as they may lead to miscarriages. You have to tell the patient exactly what to take and not take to ensure their safety.” (IDI_Female_04)

#### Factors that hindered herbalists from providing informed consent.

Various structural challenges also hinder herbalists from implementing IC processes, such as limited time at the premises of the herbalist. A unique aspect of this finding was that most female patients visited herbalist practices without their husbands’ permission, necessitating a quick return home. In view of such short visits, the women received information only about the use and dosage of medicines.

“Occasionally, patients arrive in a rush, leaving little time for extensive information about the treatment. In such cases, they are mainly interested in knowing the dosage and how to take it. A significant portion of these hurried patients are often married women who leave their homes without their husbands’ approval, necessitating a prompt return home. (lDI_Female_02)

Most participants said they lacked the knowledge, skills, and self-efficacy to practice IC and that they had not had any form of training or had heard of the IC concept or its value and obligations.

“…therefore, the question of providing adequate information has to be informed by systematic training that would lead to someone somewhere formulating an herbal product that would come with information regarding effectiveness, safety, and contraindications, which will now guide this person going to use it to inform the other user” (KII_Female_03).

Some participants did not see the meaning or importance of signing IC forms or patients having choices as to whether they accepted or refused the herbalist’s treatment. The perception that IC should be used only for very risky health procedures, such as surgery-hindered IC practices by herbalists since they do not perform major surgeries:

“Written consent is obtained in most cases when major surgeries are going to be conducted like operations… for us in herbal medicine, we do not sign these forms because we do not perform major surgeries.” (KII_Male_04)

Few participants disclosed much information to the patients because they feared losing the market for their medicines. Patients may notice that the drugs are readily available and accessible in the environment.

“If we disclose all that information to the patient, we will not attract a market for our medicines. Once we have shared all the details they need to know about their treatment, there’s little incentive for them to return to us when they can access the information on their own.” (IDI_Female_l7)

Some participants said that patients are generally not educated and have poor literacy; moreover, they do not know their rights or, thus, what to ask or expect from the herbalist.

“The majority of herbal medicine consumers lack education on what they should request or expect. However, if they were mentored, they would be more inclined to ask. Unfortunately, it would be problematic if the patent were to inquire when the herbalist lacks the knowledge to provide an answer.” (KII_Female_03)

### Support needs that herbalists reported that could enable them to effectively practice informed consent

The participants also suggested the following ways to help motivate them to implement IC in their practice: receiving training in aspects of IC and strengthening collaboration with conventional medical practitioners.

“In case the herbalists are trained on what to do, they can implement the IC practice very well because it is very good practice. (IDI-Male-19)

Participants also requested that the government strengthen intellectual property protection for herbalists’ property rights and innovations. They need to assure that their knowledge will not be stolen or misused, especially after information disclosure about the herbs to the patients.

“There is also another law that handles intellectual property. Once somebody gets to know the benefit, then they share more, knowing that their knowledge can be protected… Therefore, it is all about giving confidence regarding intellectual property to the herbalists.” (KII_Female_3)

Although adopting IC was suggested by some participants, there were concerns, on the other hand, that there is a need for it to be contextualized into our African cultural context.

“Now, in light of informed consent, I think there is need to be much more contextualized into our African setting. We should not import the Western concept to the traditional African way of living”. (KII_Male_4)

The introduction of the law in Uganda that prompts herbalists to obtain IC from their patients and build their capacity to do so would promote this practice. Participants reported that if the law obligates the practice of IC, they would abide by it if they were skilled enough and would know what to do.

“The government should enact laws that guide the practice of traditional medicine and aims to uplift and enhance the capacity of herbalists. The existence of these laws is crucial, as some individuals enter the field by emulating others without proper training. The government’s support in regulating and building the capacity of herbalists will help maintain standards in the practice.” (KII_Female_03)

## DISCUSSION

This study investigated herbalists’ practices and attitudes regarding the IC process, focusing on the application of key elements such as information disclosure, competency, understanding, and voluntariness in the context of herbal medicine. The necessity of obtaining patients’ informed consent in clinical care originated from 20th-century legal precedents resulting from cases such as *those of Mohr v Williams* and *Pratt v Davis in 1905 and from the Rolater v Strain* and *Schloendorff v Society of New York Hospital,* which further solidified the principle of patient autonomy and the right to self-determination (14–16). This requires a competent individual with the ability to understand and weigh medical information to make decisions (17) free of coercion or undue influence (18). This study revealed various sources of herbal medicine knowledge among herbalists, influencing their attitudes and practices toward IC. According to Nzaumvila et al.’s research in the Congo, among practitioners, informed consent associates their ability to exercise information disclosure with having formal training in medical ethics and IC (7). Herbalists with formal education who obtained herbal medicine knowledge through structured programs may tend to exhibit more positive attitudes toward informed consent than those with informal education, those who rely on internet sources or those who have experiential knowledge acquisition. Healthcare practitioners, including herbalists, often receive insufficient or no training in IC practices, leading to potential misinterpretations of the requirements and legal standards of IC. Looking at the different elements of informed consent, participants’ attitudes and practices were as follows:

### Information disclosure

Most participants expressed a positive attitude toward IC, emphasizing the importance of information disclosure in dispelling misconceptions about herbalists being “quacks”, empowering and educating patients to enable them to actively participate in their healthcare decisions.

These findings resonate with those of Nzaumvila et al., who examined the knowledge and practices of seeking informed consent by healthcare workers in the Congo, and Langworthy, who explored the procedures for providing consent among chiropractors in the United Kingdom. (5, 7, 19) Their results further align with Uganda’s constitutional right to information and the Uganda Patient Charter (20, 21).

At a minimum, the information disclosed should entail the diagnosis, the procedure and its risks, the benefits, and the alternatives, including choosing nothing (16, 17). The kind and amount of information shared by herbalists were determined using the reasonable physician standard, where healthcare providers (HCPs) disclose only that information that any reasonable physician would disclose to a patient in such a situation (17, 22). This kind of disclosure has been considered by some writers to limit patient autonomy. Given that herbal medicine relies on trust and patients are treated individually, adopting a subjective reasonable standard for information disclosure would be ideal. This approach tailors information based on each person’s unique needs, ensuring more patient-centered care (18, 22). On the other hand, considering that herbalists commonly perform straightforward medical procedures, can we view full disclosure of all the relevant details unnecessary? Does an incomplete discussion of these factors invalidate the consent obtained by the herbalist?

The study participants’ decision to selectively disclose information to patients may be considered reasonable for preventing social harm and potential stress to the patient and family resulting from the information provided during IC. This finding is similar to that of Jose and Alhajri, who argue that discussing side effects can alleviate patient anxieties; however, this finding differs from Akpa et al.’s findings, where traditional health practitioners unanimously supported discussing side effects with patients (23), attributing it to improve satisfaction, and assisting in informed decision-making (24). Therefore, it is crucial to strike a balance between providing adequate information and avoiding potential harm.

### Patient comprehension

Information disclosure should be followed by an herbalist to ensure adequate comprehension, although standardized methods for assessing understanding are lacking (25). Study participants reported assessing comprehension through body language, nonverbal cues, follow-up sessions, asking patients to repeat what the herbalist would have explained and question-answer approaches, and some of these have been reported to be effective methods of ensuring comprehension by patients (25). Relying on body language aligns with a patient-centered communication approach, fostering trust and reducing the risk of miscommunication. Recognizing patient understanding provides herbalists with an opportunity for immediate clarification, promptly addressing signs of confusion or uncertainty. In cases of limited patient capacity, such as children or those who are mentally unstable, herbalists obtain IC from a competent adult or proxy.

### Voluntariness and decision making

Regardless of patient knowledge level, it is reassuring that herbalists feel obligated to educate and guide decision-making through information disclosure and hence facilitate decision-making. Study participants recognize patients’ opinions and knowledge, challenging the herbalist’s role as the sole decision maker (26) and emphasizing patient autonomy. Shared decision-making (SDM) between herbalists and patients was considered ideal by participants, fostering mutual advice and education during treatment discussions and aligning with SDM’s goal of respecting, protecting, and promoting patient autonomy(26, 27). Decisions were generally guided by community or societal preferences, emphasizing the notion that societal illness is considered a communal concern rather than an individual burden, further exhibiting Ubuntuism and social responsibility. In many African cultures, family values take precedence over individual decision-making, a departure from the common medical practice of decision-making practices that tends to prioritize the individual (28). In 2007, Terry reported that in conflicts between a patient’s advance directive and family wishes, more than half of the patients preferred family decisions over their own decisions (17). Despite the recognized patients’ right to make decisions, some patients often prefer not to make decisions independently but desire SDM with family or physicians or entrusting others to decide on their behalf. In addition to the crucial role of sharing treatment information, patient decisions may also be influenced by patient-herbalist relationships and trust, particularly among referred patients familiar with the effectiveness of herbal treatments (Grady, 2015). Sensitivity to cultural values must always be an important consideration when obtaining IC.

### Documentation

Written documentation of ICs is a standard practice in medical practice and research (18). However, herbalists, as revealed in this study, seldom utilize it for IC. Verbal consent and mutual agreements are more common among herbalists than among traditional health practitioners in Nigeria (23). The dislike for and hence absence of IC forms among herbalists may stem from a lack of writing skills and the perception that signed forms are unnecessary, especially for nonsurgical procedures. This aligns with the perspectives of CAM practitioners by Caspi et al., who viewed the IC process as a legal nuisance (25, 29). Although some herbalists in this study expressed fear of legal consequences, many relied on trust-based patient-herbalist relationships in close-knit communities. The perceived impracticality and time consumption of signing consent forms also hindered the fulfillment of some aspects of the informed consent process. Participants in this study highlighted the concept of implied consent, evident in patients accepting referrals and simply visiting the herbalist, signaling agreement with the herbalist’s suggestions. Therefore, sophisticated procedures such as signing consent forms were considered unnecessary and uncommon. This type of consent aligns with common practices in healthcare, such as patients extending their arms for routine procedures such as blood drawing or blood pressure checks. In such cases, patients are presumed to be aware of the procedure’s implications, obviating the need for additional explanations (7, 18, 30). However, should we consider this kind of consent adequate in herbal medicine?

Limitations of this study include the absence of observation of herbalists attending patients, hindering an assessment of their informed consent practices. An ethnographic study could address this gap by involving herbalists and observing their daily incorporation of informed consent principles in practice.

## Conclusions

This study investigated herbalists’ practices and attitudes toward IC in herbal medicine, revealing positive attitudes toward information disclosure to dispel misconceptions, empower patients, and enhance professionalism. The study revealed the practice of selective information disclosure, stressing the need to discuss side effects of patient participation in therapy while neglecting the disclosure of alternative and herbal formulation constituents. Unique comprehension assessment methods include using patients’ body language and a shared decision-making mechanism where family and societal decisions supersede individual decision-making. The absence of written documentation on herbalists’ IC practices was identified, prompting a call for education and training within the herbalist community emphasizing the legal and ethical implications of non-documentation. This study offers valuable insights into the complexities of IC in herbal medicine, suggesting avenues for improvement, training in IC concepts, and standardization within the community through the enactment of laws and guidelines mandating the practice of IC among herbalists, including the introduction of intellectual property rights for herbal medicines to reduce secrecy that could limit IC. This highlights the need for tailored communication that balances cultural nuances while ensuring patient participation in healthcare decisions. Overall, the study provides valuable insights for enhancing communication, upholding patient autonomy, and advancing professionalism in herbal medicine.

## Figures and Tables

**Table 1:** Sociodemographic characteristics of the IDI participants.

*Population Demographics*	Number (N = 21)

***Age***	**2**
**30–40 years**	

41–50 years	7

51–60 years	5

61 –70 years	7

**Gender**	6
Male	

Female	15

**Education status**	14
Primary education	

Secondary education	6

Tertiary	1

**Experience working as a herbalist**	1
< 5 years	

5–10 years	3

11–20 years	7

>20 years	10

## Abbreviations

CAM: Complementary and Alternative Medicine
CM: Conventional medicine
HCP: Health Care Provider
IC: Informed consent
IDI: In-depth interviews
KII: Key informant interviews
REC: Research Ethics Committee
SBS REC: School of Biomedical Sciences Research Ethics Committee
TCM: Traditional and Complementary Medicine
THP: Traditional Health Practitioners
TM: Traditional medicine
WHO: World Health Organization
